# Deep neural network and model-based clustering technique for forensic electronic mail author attribution

**DOI:** 10.1007/s42452-020-04127-6

**Published:** 2021-02-18

**Authors:** K. A. Apoorva, S. Sangeetha

**Affiliations:** Department of Computer Applications, National Institute of Technology, Tiruchirapalli, Tamil Nadu, India

**Keywords:** Deep neural networks, Model-based clustering, Enron, Author attribution, Digital forensics

## Abstract

Electronic mail is the primary source of different cyber scams. Identifying the author of electronic mail is essential. It forms significant documentary evidence in the field of digital forensics. This paper presents a model for email author identification (or) attribution by utilizing deep neural networks and model-based clustering techniques. It is perceived that stylometry features in the authorship identification have gained a lot of importance as it enhances the author attribution task's accuracy. The experiments were performed on a publicly available benchmark Enron dataset, considering many authors. The proposed model achieves an accuracy of 94% on five authors, 90% on ten authors, 86% on 25 authors and 75% on the entire dataset for the Deep Neural Network technique, which is a good measure of accuracy on a highly imbalanced data. The second cluster-based technique yielded an excellent 86% accuracy on the entire dataset, considering the authors' number based on their contribution to the aggregate data.

## Introduction

As "Words are mightier than action," people worldwide use some textual form of communication as an oral way of communication isn’t always possible. There are other textual communications used by people to share formal or informal messages. Gone are the days when people used written letters to share information with others, which would take a lot of time. As technology invaded us, sharing of information also happened with ease and at a faster rate. The sharing of data with the Internet's help became very common and is termed as an Email (Electronic mail) mode of communication and forms significant documentary evidence in digital forensics. To mention a few, the other forms of documentary evidence are online messages, posting of information on social media, e-articles. Emails are used to share formal letters, such as scheduling meetings in the corporate world, business communications, online reservations and presentation calls, to name a few, while informal emails include personal greetings and family invitations. The above mentioned are the actual usage of Email.

Nowadays, Email is used for many illicit activities, ranging from cyber terrorism to cyber war to many other cyber scams. Cybercriminals are making use of Email as a primary source to target many attacks. The reason is it hides the individual's identity by sending anonymous fraudulent emails to several recipients at one instant. The most popular types of acts performed using emails are sending spam emails, which are meant to obtain personal information of the recipients through messages as swindling contests and prize-winning advertisements. The other popular type includes phishing, which includes a fraudulent link in the Email and once clicked drags the recipient to a fraudulent site, which extracts the personal information. Few popular email attacks where Yash Raj Films got defrauded were two fraudsters pertained to be costume designers and lobbed and Rs. 1.75 lakhs [22]. A recent attack in mid-march 2020 targeted world health organization (WHO) because of the COVID-19 crisis, where the attackers try to lure agency employees into logging on a phishing site and handing over their login credentials [23–25]. Apart from the attacks mentioned above, many IT companies are also targeted by the email racket scam [26]. Emails were used in the 9/11 twin tower blast, where the report revealed the terrorists shared several emails before the crash. There were few pieces of evidence of emails connected with the Mumbai attack in 2008 [27]. There is also a recent email ploy in these COVID-19 pandemics where the hackers linked to Pakistan disguised themselves as the Indian government sending malware emails. When the user clicks on the Email link, any sensitive information about credit cards, passwords and location details are transferred to the hacker [33].

They recorded and experienced lots of email scams, which paved the way and stimulated us to find the authors of the shared emails using a different machine and deep learning techniques. With the rise of on toward incidents happening, there is a need for textual email forensics. There are two broad types of email analysis: (1) One using the email header information) (2) Using the Email body. Our work focuses on type ii, which uses the email body, which has textual information. Each Email is written by an author who has unique writing styles. Every person has unique writing styles known as author stylometry. Based on the author's stylometry, identifying the actual author of the email pitches in. The work started early in 1887 when Thomas Corwin Mendenhall analyzed authors Beacon, Marlowe and Shakespeare to distinguish their writing styles from one another. Each person has a unique style of writing. Hence, a model that predicts the correct author of the text based on a writing style is essential.

In this paper, we propose a deep neural network (DNN) and cluster-based classification technique for email author attribution. First, we vectorize the data and use DNN architecture on different author texts, which yielded different accuracy rates. Second, clusters were framed, which included authors based on 1% of their contribution to the entire dataset. Then classification technique Naive Bayes was applied to the groups. Following are the contributions of the paper:

1. Adaption of deep neural network and model-based cluster classification based on author contribution towards the entire dataset consider for the analysis.
2. Achieved high accuracy of 86% on considering a large number of authors of 149.

As the proposed model attains a good accuracy considering the stylometric features of authors, it had its applicability in investigating insider threats and other fraudulent activities where the emails are shared with the same community.

## Dataset

The Enron email dataset [21] was prepared by the CALO project (a cognitive assistant that learns and organizes). It has an extensive collection of about 150 users' real emails, most of Enron's senior management organized as folders. The data contains around 0.5 M messages. The information was publicly made available on the web by the federal energy regulatory commission. Enron email dataset is a benchmark dataset used for experimentation for various email researches.

## Problem statement

We define our problem as identifying the author of an email based on the stylometric features set. Mathematically, the problem is represented as follows:

1 $$\left\{ {e_{1} , \, e_{2} , \, e_{3} - - - - - - - - - \, e_{n} } \right\} \, E$$

2 $$\left\{ {a_{1} , \, a_{2} , \, a_{3} - - - - - - - - - \, a_{n} } \right\} \, A$$

3 $$\left\{ {f_{1} , \, f_{2} , \, f_{3} - - - - - - - - - \, f_{n} } \right\} \, F$$

4 $$\forall E \, \exists A \mathrel\backepsilon F \, = \, E_{i} \overset{\wedge}{=}A_{j}$$

Equation 1 represents E as a set of emails in the dataset, Eq.2 A is the set of authors of Email in the dataset and Eq. 3 F is a set of unique features that the author possesses. Expression 4 says for all emails in the dataset; the actual authors have unique features, which serve as a mapper of Email E_i_ to author A_j_. During the training phase, emails of the author are analyzed and features are extracted. At the testing phase, emails are checked against these extracted features and prediction accuracy is calculated.

The rest of the paper is organized as follows: The related work in Sect. 4, the proposed model, is presented in Sect. 5. The experimental model and discussions are described in Sect. 6, the conclusion and future scope in Sect. 7.

## Related works

This segment reviews the works related to the authorship attribution/ identification based on the content analysis. The content analysis focuses on textual information or the writings of different authors. Authorship Identification is the process of finding the author of a text from a group of anonymous documents or books. Authorship attribution or Identification is considered the text classification problem in which authors are regarded as classes and their writings are considered text to be classified. Various usages of machine learning and deep learning techniques are applied for the author classification problem, where the work is to predict the actual authors of the considered text.

Our work focuses on the authorship attribution problem based on the email content analysis. An email consists of header and body sections where the header details are stripped off and the body of the Email alone is considered for the study. Here authors are regarded as classes and the body of the email written by the respective authors is the text to be predicted.

The authors [11] presented a holistic analysis of the email classification task. The techniques were supervised, semi-supervised, unsupervised machine learning techniques, statistical learning and content-based learning. Based on the various primary machine learning techniques mentioned, the authors also highlighted the multiple algorithms used in each machine learning technique based on which the email classification process can be performed. The survey provided a detailed analysis of various email classification techniques and presented the research advances in each email category.

The work conducted by Ahmed M. Mohsen et al. [2] considered the email author attribution using deep learning techniques. Techniques as stacked denoising autoencoders [SDAE] for feature extraction from documents were used and then a support vector machine classifier was used for classification. The corpus used in this work is a subset of Reuters Corpus Volume 1. It is labeled according to the author's writing. Only a small set of 50 authors were considered for the study and it was a class-balanced dataset as each author in the corpus had written 100 documents. When compared with the previous workings, they achieved promising results with reasonable accuracy. They considered performing feature normalization, feature selection to remove redundant features and retain features with significant info gain in each category. Feature extraction is of prime focus for arriving at good results. They achieved an accuracy of 95.12% over a set of 50 authors of balanced class.

The research conducted by Nilan Saha et al. [3], where author identification for short texts was considered. The technique used here was multi-layer perceptron (MLP). The Twitter testbed was used for the study purpose. The work was conducted considering a small dataset comprised of 20 users who had published more than 400 tweets. Tf-idf vectorizer was used for extracting linguistic features. The accuracy measure was 96% for four authors with 195 tweets, but as the number of author’s increases, there was a gradual drop in the accuracy measure, with 67% for 20 authors.

The analysis conducted by Naser Eddine Benzebouchi et al.[4] stressed the work of the email author identification system using a text representation vector as word2vec. The result was performed as a two-stage process. The first stage emphasized the unique feature extraction from the raw document using word2vec. The second stage is applying a multi-layer perceptron (MLP) classifier using a back propagation learning algorithm. The work was conducted on a pretty small dataset consisting of 8 authors with nine documents for each, where-in, where the authors used six records for training and the remaining 3 for testing. The authors used PAN 2012 English dataset for the research. They achieved an accuracy of 95.83%, considering eight authors.

Ekin Ekini et al. [5] provided a study on ensemble classifier usage for analyzing the emails to identify the authors. Bagging and AdaBoostM1 algorithms were used as ensemble classifiers, which rendered a fair accuracy of nearly 81%. They considered a small dataset of 5 authors, which contained 250 emails from which 49 textual features were extracted.

The authors Luke Chen et al. [6] proposed a collection of author attribution models that identified the authors of limited texts from Twitter messages. natural language techniques (NLP) techniques as Tokenization and Lemmatization were used to extract lexical, syntactic and semantic features, which were then given as input to the classifiers Naïve Bayes, support vector machine (SVM) and neural network multi-class classifiers. The authors achieved promising results with 89% classification accuracy, where they considered a minimal set of 6 authors, each with limited Twitter messages.

The research projected by the authors Chen Qian et al. [7] considered the two problems of author identification and verification. Two datasets used for their work are Reuters 50–50, which consists of archiving category data based on newswire stories. Top 50 authors were considered for the study. The other dataset used was the Gutenberg story dataset, which was collected and labeled by the authors. Top 50 authors were considered with each author's contribution of around 900 paragraphs from their stories. The authors achieved an accuracy of 69.1% on the C50 dataset and 89.2% on the Guttenberg dataset for email author identification. As the work experimented on a pretty small set of authors, they achieved 99.8% accuracy for author verification on both datasets.

The stylometric approach was applied by the authors Hoshiladevi Ramnial et al.[8] to demonstrate how author identification can be used to identify plagiarism in various formal writings. The dataset used for their work consists of a computer program with 44 features and ten Ph.D. theses split into different segments of 1000, 5000 and 10,000 words, which lead to the corpus of 520 documents. They arrived at an excellent score of over 90% with 10,000 words, but the score gradually decreased to around 73% with 1,000 words. The authors demonstrated their work, increasing the number of authors gradually from 2 to 10. An increase in the number of authors decreased the accuracy measure as well. The authors employed k-Nearest Neighbor (k-NN) and sequential minimal optimization (SMO) learning algorithms for their demonstration.

The authors [9] applied the author identification process slightly, whereby applying the concepts of motifs for the identification task. Features were extracted from the co-occurrence networks and were used as classification features. Four learning algorithms as C4.5, k-NN, SVM and Naïve Bayes were used for the process. They obtained results that were similar to the chase baseline score of 12.5%. The best result of 57.5% was obtained on a dataset of 8 authors, which comprised 40 novels from the project Gutenberg repository. However, the work got was less than the other traditional approach, which had an accuracy rate of 65%. The authors mainly highlighted network motifs in their work and suggested further exploration of other linguistic tasks.

The use of stylometry has been an essential aspect in the field of classification problems. Similarly, it has varied usage across different domains. The stylometry approach used by the authors Justin Zhan et al. [10] emphasized a short text category classification problem, using deep neural network techniques for semantic enrichment. The authors experimented on, with and without the inclusion of nouns and verbs in the texts. The experimental results were fruitful with suitable accuracy measures on a smaller dataset of news titles with seven categories.

A supervised framework for author identification was proposed by the authors Shanta Phani et al.[12], The authors concentrated on the lexical and shallow features of the author's writing styles. They explored the possibility of using the topic-modeling-inspired elements on the documents written in Bengali to classify them according to their actual authors. The corpus consists of 3,000 disjoint literary passages written by three eminent Bengali writers. The authors considered authorship attribution as a classification problem. High accuracy was achieved using seven different classifiers as Bernoulli Naïve Bayes, Multinomial Naïve Bayes, Decision tree, Random Forest, SVM Support Vector Classifier (SVC), SVM Linear SVC and Logistic Regression on the considered dataset of only three authors.

The authors [13] explored the author identification problem on the Lithuanian language datasets, which id of 2 different domains: formal and informal writing. Legal writings were the parliamentary data and casual includes the forum posts. The authors achieved 70.6% accuracy on 100 authors, which exceeded their baseline score of 62.7%. The authors also experimented with the process considering different numbers of authors ranging from 3, 5, 10, 20 and 50. The authors employed two supervised machine learning techniques-SVM and Naïve Bayes multinomial (NBM) for the classification task to explore further findings.

The authors [14] applied the authorship attribution problem and verification differently by following a graph-based approach, namely an integrated syntactic graph. Features were selected from the shortest path traversal through the combined syntactic graph. The authors also showcased the patterns identified with their method, which can be used for various document analysis tasks. The authors considered unsupervised techniques for the process and achieved a 68% score on C10 corpus, which consists of 10 authors, each with 50 documents. The authors also evaluated their model on the PAN'13 data and achieved a score of 83.3%.

The authors [15] made a comparative analysis in classifying the tweets based on their respective authors. The authors used logistic regression and Naïve Bayes classifiers for their evaluation. Around 46,895 tweets were taken into consideration for the task. As tweets were only made up of 280 characters finding a perfect technique for the authorship classification were a challenging task. The authors extracted unique features from the tweets with different NLP techniques as Bag of Words and vector space model. Once removed, the feature vectors were fed to the machine learning classifiers for author identification. The authors achieved promising results with 91% accuracy on logistic regression and 90% on Naïve Bayes classifier.

The authors [16] Paolo Rosso et al. presented a model for the authorship attribution task. The authors presented their evaluation as a comparison on different techniques as on Convolution neural network (CNN-1) and CNN-2, Long Short Term Memory (LSTM), Schwartz et al. work (SCH) [20], Character and word n-gram (CHAR). Their experimental results showed that CNN over n-grams performed well compared to other techniques that achieved 76% accuracy over a small set of authors with 1,000 tweets each. However, the accuracy score gradually decreased with an increase in the number of authors. For 100 authors, CNN achieved around 50% accuracy, 200 authors to 48%. The authors evaluated the accuracy score better with an increase in the number of tweets per 50 authors, around 72% for 500 tweets.

The authors [17] approached the problem of authorship attribution of small-texts posted on social media platforms using the concepts of user-specific stylometric and Onomatopoeia that deals with the formation of original words used for the specific or poetic purpose. The authors used the convolution neural network for the author's classification task. The English twitter corpus, which consists of 128 million small tweets from 50,000 users, was collected for the study. The author's employed short texts or micro messages on the social media platform as unidimentional signals. The texts from authors were considered only once where-in the re-tweets were ignored. The authors achieved good training accuracy of 97.5%, but the validation accuracy was dropped to 65% but was close to the baseline accuracy.

The authors [18] Ankita et al. approached the authorship attribution problem considering the stylometric approach of authors using multi-layer neural network and SVM machine learning techniques for classification tasks also employed respective voting systems for their work. The dataset used for the study is language-specific consisting of recent eight Bangladeshi bloggers. A web crawling mechanism collected the data. The corpus consists of a total of 1,765 Bengali articles. The authors achieved good experimental results with an average accuracy of 76.4%, 75% on classification models, 81.7 and 85.13% for voting systems.

The authors proposed a cluster-based classification technique [19] for email authorship attribution on the Enron dataset with a slightly higher number of authors considering 10, 25 and 50, where promising results were obtained. In this study, the authors achieved an accuracy of 94.3% on ten authors, 89% for 25 authors and 81.3% for 50 authors. The results were quite promising when compared to the earlier works of Iqbal et al.[1] The experiments were conducted on 20 authors and the accuracy was less than 75%.

We propose a double approach, where a comparison was made against the earlier works on email authorship identification. At first, a deep neural network with a Term frequency-inverse document frequency (Tf-idf) vectorizer was used and the experiments were conducted on 5, 10, 25 and the entire unbalanced dataset. The accuracies were pretty good, ranging from 94% for five authors, 90% for ten authors, 87% for 25 authors and 76% for the entire dataset. Second, we worked on the cluster-based classification technique where the whole dataset was considered for the study. The authors were split into 'k' clusters, depending on their contributions. The authors, with at least 1% of contributions, were considered. Once the clusters are formed, we have applied the classification techniques like Naïve Bayes and SVM on the clusters and achieved an accuracy of 85% on the entire dataset with tenfold cross-validation, which is quite encouraging. The purpose of using a double approach is to make a comparison of the efficiency of models considered.

## Methodology

**Fig. 1:**
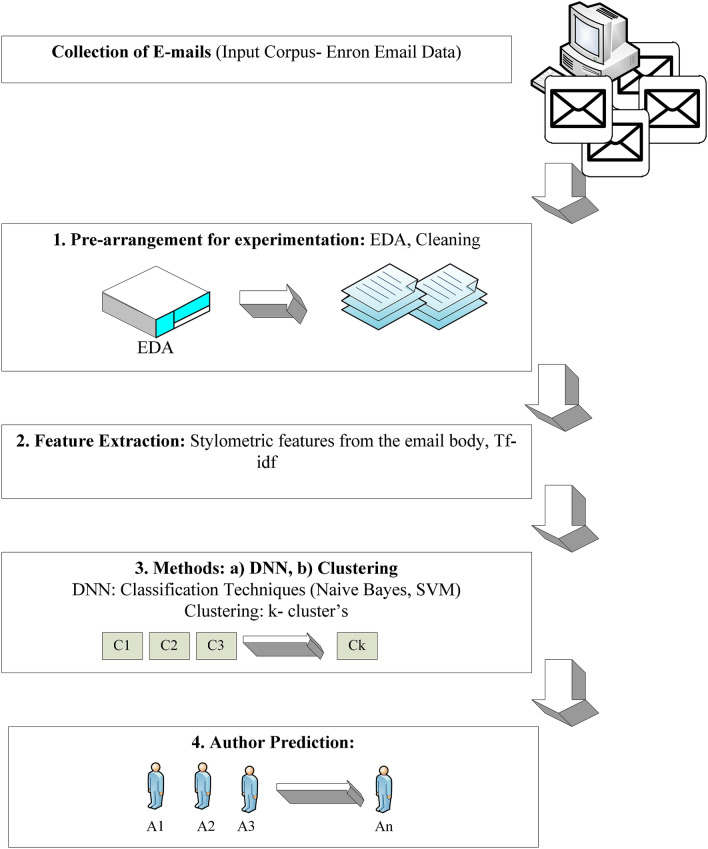
Author prediction process

The general idea of our method is depicted in Fig. 1, which is a sequential process comprising four steps: 1. Pre-arrangement: an initial exploration of the data is performed to know the statistics of the data. Next, the email body is extracted. 2. Feature extraction: The stylometric features of authors are extracted and are vectorized for further subsequent processing. 3. Methods: deep neural network (DNN) is used along with the machine learning classification techniques and a clustering method is used to show the efficiency in the authors. 4. Author Prediction: based on the methods used, the authors of the Email for the written content are predicted.

### Pre-arrangement

In the Enron email dataset, which is unbalanced, an initial exploration of the data is performed to know the statistics of the authors' contribution. The next step is to emphasize the email body as our work focuses on the content analysis of Email. The email headers are stripped off and each author's email body based on all folders considered is taken into consideration and is stored in a CSV file for further processing.

### Feature extraction

The proposed model uses machine learning techniques for clustering and classifying emails to respective authors; the essential aspect for this to work is to create a feature set. Each author has a unique way of writing and presenting the information that forms the authors' actual writing style.

**Fig. 2:**
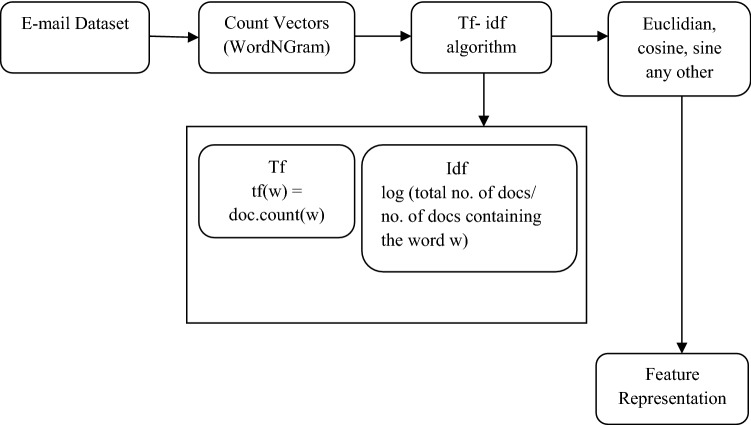
Tf-idf working

Based on these facts, the stylometric features are extracted for each author considered in the entire email dataset. These features are extracted from the email body's content. The weights are assigned using a statistical measure tf-idf vectorizer (Fig. 2), which evaluates how similar the word is for the document in a collection of documents considered. Tf-idf is a popular algorithm to transform the text into a meaningful representation of numbers or vectors'. The representation depicts the significant characteristics of a text. Tf-idf is an occurrence-based vector representation with a combination of two measures Tf (term frequency), which assigns more weights to a term in an email that occurs in many other authors through normalizing the occurrence of the term with the size of the specific email body; this serves as a discriminating factor in differentiating the emails from various authors.

The cleaned email dataset is passed over the count vectorizer, which has the word n-gram Analyzer, which normalizes and lemmatizes the data and prepares it for the tf-idf application. The term frequency and inverse term frequency are calculated and with the help of a few similarities.

measure as Euclidian, cosine the useful features are represented numerically.

5 $$tf \left(w,e\right)=\frac{freq(count\left(w\right),e))}{|e|} =>tf \left(w,e\right)=\mathit{log}(1+freq \left(w, e\right))$$

6 $$idf \left(w,e\right)=\mathit{log}\frac{|E|}{cont(e \in E:w \in e)}$$

7 $$tf \left(w, e, E\right)=tf\left(w, e\right)\times idf (w, E)$$

where $freq \left(w, e\right)$ returns the frequency of the term in the email e, |e| is the total number of words in the email body, $tf \left(w,e\right)$ returns the normalized frequency of the term t in email *e*. $idf \left(w,e\right)$ returns the inverse document frequency, which returns 0 if the word is prevalent and appears in many documents or one otherwise. |E| is the total number of emails in the complete dataset. It $count (e \in E:w\in e)$ returns the number of emails containing the word w in the email body. Finally, the product of Eqs. 5 and 6 returns the more important word in the Email, which is Eq. 7.

Following are the features considered for our work:

Number of words in the text.

Number of unique words in the text.

Number of characters in the text.

Number of stops- words in the text.

Number of punctuations in the text.

Number of upper-case words.

Number of the title- case words.

The average length of the words.

### Methods

In our proposed word, we have emphasized the usage of DNN and clustering, which yielded promising results for author attribution compared to previous related works.

#### DNN (deep neural network)

An artificial neural network (ANN) is an information processing paradigm and is inspired by the biological nervous system's working as the brain processes the information. It comprises highly connected processing elements called neurons that work in unison to solve a particular problem. When many layers of artificial neuron connections are used, it is termed as "deep neural network" or "deep learning." Our approach focuses on using dense layers that were already proposed for stylometric characters [28]. We intensify this approach with our features considered as each author has unique writing styles.

The mathematical form of each hidden layer is of the following form:

8 $$y=Wx+b$$

*X* is the input data (for the first layer), *W and* *b *is the weight and bias matrix are considered parameters that are learned during the training process. As all the neurons are connected in the network, it is called dense layers.

Each node also encompasses an activation function that plays a vital part in any neural network as it determines the output of a neural network. For our work, we have chosen relu and softmax as it performed well for our task.

9 $$\mathrm{z}=\mathrm{f}\left(\mathrm{b}+\mathrm{e}.\mathrm{w}\right)=\mathrm{f }(\mathrm{ b}+ \sum_{\mathrm{i}=1}^{\mathrm{n}}{\mathrm{e}}_{\mathrm{i}}.{\mathrm{w}}_{\mathrm{i}})$$

Equation 9 represents a function for a node in the network; the weighted sum of inputs *e *is passed over an activation function and bias *b*.

Data moves across all connected nodes in the neural network and gets activated with the activation function's usage. At the last layer, the authors get assigned to the data, which are the predicted values.

Training consists of an iterative run through all the entire dataset by assigning weights and biases. The probabilities for each author are collected and gradients are calculated for all parameters. The gradients are the vectors pointing to the local minima of the loss function. We have employed a categorical cross-entropy loss function. The global minima are used to optimize the entire network; we have used Adam optimizer designed for an in-depth neural network process, which finds the learning rates for each parameter. We have used this optimizer as it outperforms on handling sparse gradients on noisy problems. My dataset considered for the study has the most common features and working with the Adam optimizer proved to yield good results.

Our approach has created a three sequential neural network and has used fourfold cross-validation for testing accuracy. On increasing the number of folds and epochs, the accuracy can further be enhanced.

#### Clustering

Clustering is an unsupervised learning method. It refers to dividing the dataset into different groups or clusters, such that each group has high intracluster similarity with low inter-cluster similarity. Concerning our email author attribution task, emails in one cluster will have to adhere to an incorrect prediction of present Email in a group compared to emails in another cluster.

In specific, there are no such criteria for forming the number of clusters; it depends on the user to specify which criteria satisfy their task. There are few different methods employed for creating cluster as the density-based clustering [29], where other size clusters containing noise and outliers can be formed, model-based clustering [30] where the data is viewed as from probability distribution, the fuzzy clustering essentials [31] this maps an element to be present in more than one cluster, hierarchical k- means clustering [32]; this works explicitly on improving the results of k-means.

We have used model-based clustering, which makes a soft assignment of values to specific clusters depending on the data point probability to which cluster it belongs. We have employed this on the entire dataset considered for the task. The authors are put into different clusters based on their probability of contribution. Each cluster will have other volumes and shapes. Our study provided promising results as compared to the methods employed in literary works. As the dataset considered consists of numerous emails based on the author's contribution, we achieved excellent results with 21 optimal clusters for our task, with each models consisting of the following number of authors 6, 5, 4, 5, 7, 4, 10, 5, 7, 8, 4, 13, 8, 10, 5, 8, 8, 12, 6, 4, 4, where each cluster consists of different volume, shape and identity. The accuracy yielded 86% on the entire dataset, while most of the previous works yielded less precision on the increase in the number of authors considered.

### Author prediction

Each author has unique writing styles that decide their respective stylometry. Based on the above experimentation process of content analysis of the email body, the authors get predicted.

## Results and discussion

**Fig. 3:**
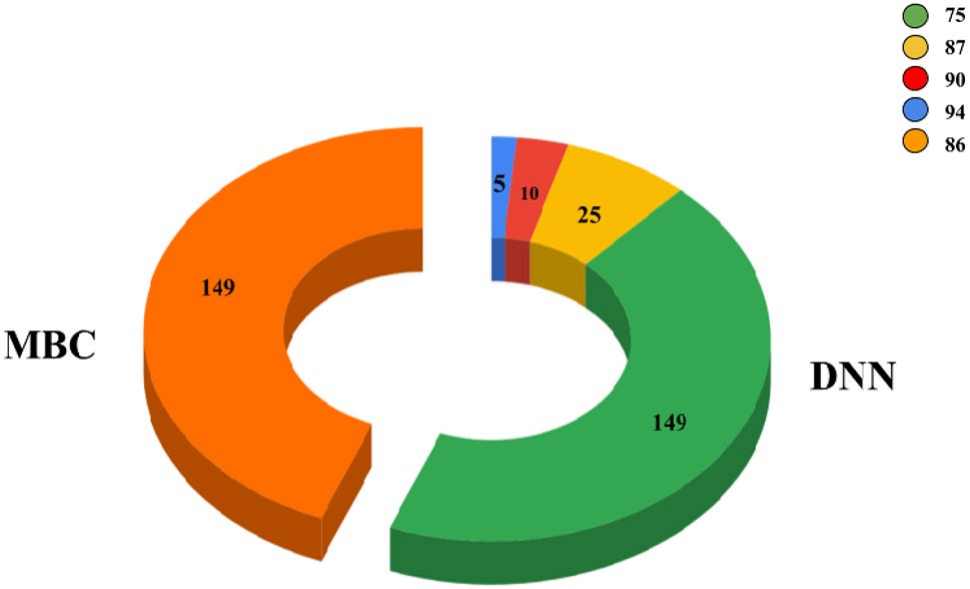
DNN and MBC accuracy: number of authors versus accuracy (%)

The working and experimental results for the DNN and Cluster-based methods employed in our work have yielded promising results. For DNN, the experiments were conducted initially on five authors then gradually increased the number of authors to check the behavior. Our work considered the application on 5, 10, 25 authors' and we have tested on the entire dataset with 149 authors. As in Fig. 3, the results depict reasonable accuracy rates on a varying number of authors'.

**Fig. 4:**
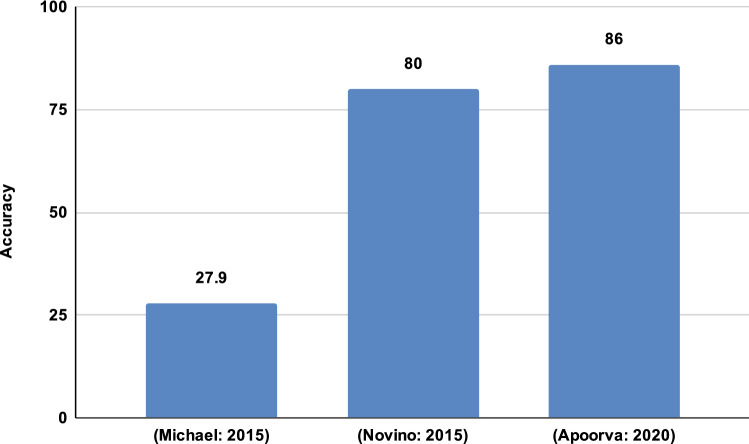
Comparison of results of authorship attribution for 149 Email authors

The literary works emphasized the application of various machine learning models along with Hierarchical clustering with less number of authors for prediction, which did not yield promising results as shown in Fig. 4, hence as stated in the introduction section, this did our work to take forward by considering the application of deep neural networks along with model-based clustering technique applied over the entire benchmark dataset considered for analysis which yielded promising results and better prediction of email authors.

**Fig. 5:**
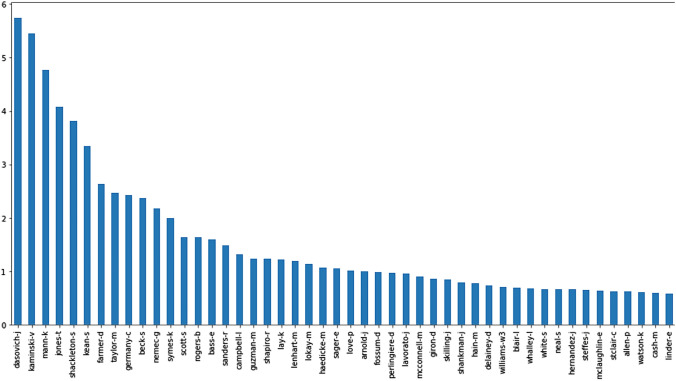

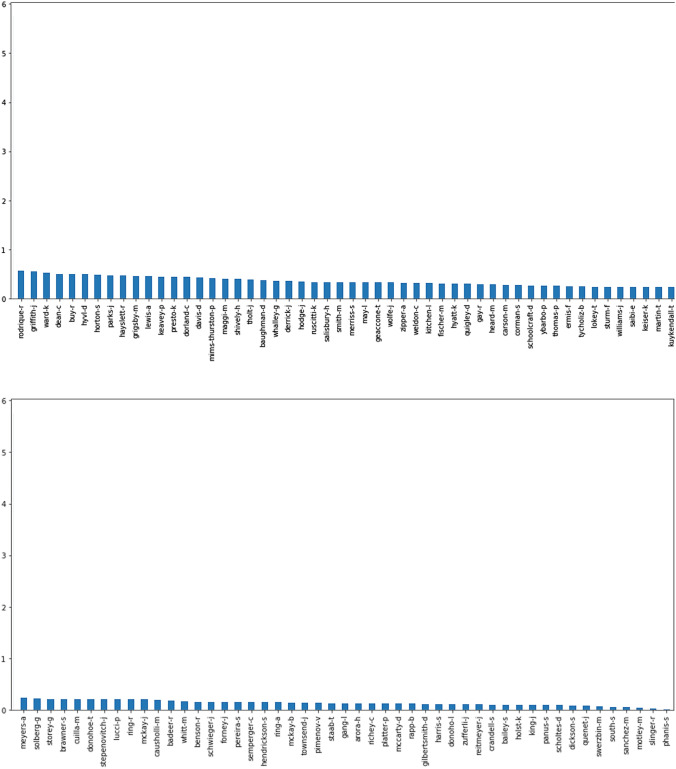
Enron author contribution

As the accuracy of the entire dataset is reduced drastically, we employed model-based clustering, where-in based on the author's contribution; they are divided into optimal clusters, which results in promising results compared to previous works. Figure 4 depicts the authors' spread and contribution to the entire dataset, where the x-axis represents the authors and the y-axis the probability of assistance (Fig. 5).

Table 1 below depicts each cluster/ model accuracy based on the author's contribution to the entire dataset. The values also show the effectiveness of the model fitting on the various clusters.

**Table 1:** Models accuracy

Model	Accuracy	Precision	Recall	f1
0	0.927017	0.920722	0.923297	0.921773
1	0.947864	0.947360	0.947066	0.947183
2	0.967156	0.965992	0.965595	0.965786
3	0.949830	0.949434	0.950767	0.949983
4	0.930995	0.930835	0.930911	0.930840
5	0.976223	0.976273	0.975988	0.976019
6	0.907955	0.908909	0.908160	0.908131
7	0.945946	0.946298	0.946415	0.946192
8	0.896905	0.896641	0.897240	0.896780
9	0.864916	0.866258	0.663792	0.864241
10	0.880356	0.680411	0.879856	0.880054
11	0.829568	0.832298	0.830493	0.830573
12	0.813433	0.815765	0.816433	0.815371
13	0.809672	0.807634	0.806936	0.806685
14	0.821186	0.816771	0.816571	0.816591
15	0.776978	0.778830	0.773543	0.773048
16	0.764748	0.757129	0.758378	0.756290
17	0.744491	0.743237	0.740560	0.741344
18	0.692868	0.692296	0.693162	0.692053
19	0.822674	0.828113	0.813128	0.817673
20	0.844156	0.792310	0.803829	0.797603

Table 2 depicts an emphasis on the overall accuracy achieved in applying the Model-Based Clustering technique.

**Table 2:** Overall accuracy of model-based clustering

	Accuracy	Precision	Recall	f1
Mean	0.862616	0.859691	0.859149	0.858772

## Conclusion and future work

This paper employed the two models for email author identification/ attribution on the publicly available benchmark Enron dataset. The authors' contribution is a two-step process i) The usage of Deep Neural network (DNN) ii) The applicability of model-based clustering. For the first method, we achieved excellent results with 94% accuracy on five authors, 90% on ten authors, 87% on 25 authors and 75% on the entire dataset. These results are comparably good to the other models used. As it is observed that on increasing the number of authors, the accuracy dripped, so we employed another method to overcome this problem, where-in by the usage of model-bed clustering, we achieved an overall accuracy of 86% on the entire Enron dataset, which is optimistic as compared to literary works. At present, our model focuses on the email author attribution task for English email content, the future work can be extended to other languages and the author verification task could be employed, which detects the untold authors as unknown.
